# Inspecting Vulnerability to Depression From Social Media Affect

**DOI:** 10.3389/fpsyt.2020.00054

**Published:** 2020-02-21

**Authors:** Lucia Lushi Chen, Christopher H. K. Cheng, Tao Gong

**Affiliations:** ^1^ School of Informatics, University of Edinburgh, Edinburgh, United Kingdom; ^2^ Department of Social and Behavioral Sciences, City University of Hong Kong, Hong Kong, Hong Kong; ^3^ Centre for Linguistics and Applied Linguistics, Guangdong University of Foreign Studies, Guangzhou, China; ^4^ Educational Testing Service, Princeton, NJ, United States

**Keywords:** depression, affect, social media, Weibo, stressful life events, rumination

## Abstract

Affect describes a person’s feelings or emotions in reaction to stimuli, and affective expressions were found to be related to depression in social media. This study examined the longitudinal pattern of affect on a popular Chinese social media platform: Weibo. We collected 1,664 Chinese Weibo users’ self-reported CES-D scores *via* surveys and 3 years’ worth of Weibo posts preceding the surveys. First, we visualized participants’ social media affect and found evidence of cognitive vulnerability indicated by affect patterns: Users with high depression symptoms tended to use not only more negative affective words but also more positive affective words long before they developed early depression symptoms. Second, to identify the type of language that is directly predictive of depression symptoms, we observed ruminations from users who experienced specific life events close to the time of survey completion, and we found that: increased use of negative affective words on social media posts, together with the presence of specific stressful life events, increased a person’s risk of developing high depression symptoms; and meanwhile, though tending to focus on negative attributes, participants also incorporated problem-solving skills in their ruminations. These findings expand our understanding of social media affect and its relationship with individuals’ risks of developing depression symptoms.

## Introduction

Depression has become one of the leading causes of disability worldwide. In 2015, 7.5% of depression patients lived with disability (1). However, many people living with this condition are not aware of their illness. In some cultures, it is also considered shameful to disclose one’s mental health problems to family members or even doctors, partially due to the tradition of presenting a positive self-image to others (2). Similar to many other countries, China is facing a severe shortage of mental health professionals; China has 23,000 psychiatrists—about 1.7 for every 100,000 participants (3). The social stigma related to cultural and moral beliefs also deters people in China from seeking treatment. Therefore, it has become essential to help individuals be aware of their symptoms so that they can decide when to seek support from professionals. In the last decade, researchers have begun to explore the possibility of using digital footprints to monitor social media users’ depression symptoms because social media data have provided a record of users’ emotional and behavioral patterns. In this work, we introduce a new approach to analyze the affect patterns disclosed on social media posts and explore how the social media affective language is associated with depression symptoms.

In psychology, affect refers to nonreflective feelings towards stimuli, e.g., the feelings of pleasure or tension (4, 5). Reduced positive affect (PA) and increased negative affect (NA) have been found to be classic markers of major depressive disorder (MDD) (6, 7). Increased NA signals negative interpretation bias and negative repetitive thinking, and decreased PA indicates a reduction of interest or pleasure in response to stimuli.

Existing empirical studies often examine people’s affect using measurement scales or professional interviews (8). The emergence of sentiment analysis provides an alternative way to study affect. Sentiment analysis is a process of extracting affective words or phrases from text. It has been found that affect, especially NA expressed in social media text, reflects social media users’ psychological characteristics and mental health status (9–11). NA is a summary of a variety of negative emotions, including anger, sadness, fear, etc. Findings from both empirical studies and social media data have shown that users with high depression symptoms tend to use more words/phrases containing NA (12, 13).

Why does the use of NA words relate to depression symptoms? On the one hand, depressed individuals tend to have cognitive vulnerabilities, which are cognitive processing biases in attention, memory, interpretation, and repetitive negative thoughts (14). For example, “No one cares about my problem,” is a negative cognitive bias. On the other hand, frequent occurrences of NA reflect dysfunctional stress response. Individuals with a dysfunctional stress response system often fixate on the causes, consequences, and meanings of stressors, which results in “stress-reactive rumination,” a passive comparison of one’s current situation with some acknowledged standard (15).

Contrary to NA, PA is in general beneficial to health and cognitive function (16). Studies have found that nondepressed individuals are often motivated to downregulate negative emotions and upregulate positive ones (17, 18), but depressed individuals usually experience reduction of pleasure (19). Similar to the empirical findings, social media users also tend to post positive content to seek social approval and/or form positive impression (20, 21). However, Locatelli and colleagues have recently found that people with depression symptoms also use more positive affective expressions in social media (19). Accordingly, they hypothesize that the relationship between PA and depression symptoms is possibly mediated by rumination.

Although there exists a large amount of evidence to support that affect expressed in social media texts can reflect mental health status, few of the studies examine the life stressors that may trigger NA and the fixation behavior. In addition, although NA has been extensively studied, there is a very limited amount of literature that examines the relationship between PA in social media content and depression symptoms. In order to fill these gaps in this line of research, this paper investigates the patterns of positive and negative affect, as well as the rumination language following a stressful life event, targeting a popular microblogging social media website in China: Weibo.

Examining life stressors presents a challenging question: What kind of events are considered to be more stressful, as opposed to those that are less so? Some stressors are uniformly perceived as more damaging to mental health than others. By asking participants from diverse cultures to rate how much readjustment was required for 42 life events, Masuda and colleagues identified a set of life events that were perceived as detrimental to mental health (22). Among others, death of a spouse, divorce, and marital separation were ranked as the top three events requiring the most life readjustment. Later studies found similar rankings in the life events requiring much life adjustment, but different rankings in those requiring moderate to low levels of adjustment, e.g., being “fired” from work dropped from the 8th in (22) to the 47th in (23). Here, we focus on three life stressors that respectively bring severe (e.g., marital separation), medium (e.g., severe illness of a family member), and low (e.g., being fired from work) levels of impact to mental health.

In light of the above discussions, this paper aims to address the following two research questions:

1. RQ1: Does social media affect reflect cognitive vulnerability to depression symptoms?
2. RQ2: What are the characteristics of stress-reactive affective content on Weibo? In particular, we focus on rumination (NA), and investigate what stressful life events people tend to ruminate on social media.

For RQ 1, we first visualized the positive and negative affect patterns of Weibo users in multiple time windows. Then, we examined the relationship between social media affect and depression symptom scores when specific life stressors were presented to the users.

For RQ 2, we examined the rumination in postevent reaction. Stress reactive rumination reinforces the interpretation bias of an individual, thus putting an individual at higher risk of developing depression symptoms. We looked into how social media users ruminated on specific life events and summarized the characteristics of the rumination.

### Contribution

This paper provides an opportunity to advance the understanding of how positive and negative affect reflects cognitive vulnerabilities to depression. By examining the stress reactive affective language on social media data, we seek to identify affective content that links to cognitive vulnerability. Addressing these issues would help better understand the pattern of affect in a social media text and its association with people’s vulnerability to affective disorders in general. Moreover, by observing what types of stressors social media users tend to ruminate on, this paper offers essential insights into cognitive biases in social media data, thus promoting future research on life events and affect in the social media context to take into account these biases.

## Data and Data Collection

### Weibo

Weibo is a social media platform where users can publish a short piece of text, video, or photo to customized lists of friends or followers. Before 2016, users could write up to 140 words on each post. Since then, the maximum number of words per post has been increased to 2,000. In Weibo, users can follow or unfollow others, like or dislike others’ posts, make comments to those posts, or share some of those posts to their social networks. By 2017, there were nearly 300 million users on Weibo, accounting for one fifth of the population in China (24). Multiple survey studies show that the majority of Weibo users are in their 20s and 30s (25, 26). Female users are more likely to mention they were diagnosed with depression than male users (27). In this study, we collected a sample of participants’ Weibo posts, and assessed their depression symptoms using a depression symptom screening test and subjective stresses of daily lives.

### Data Collection

We posted a recruitment notice for this study on a personal Weibo account on June 10, 2016. The survey was open from June to September, 2016. A few science bloggers and entertainment bloggers reposted our recruitment notice voluntarily. We also promoted our survey with paid advertisements to increase participation rates while the survey was open. The study targeted users residing in China, aged over 18. Participants of the study were asked to complete a survey containing the Center for Epidemiologic Studies Depression (CES-D) scale and a stressful life event survey. Participants could optionally sign a consent form (see Appendix A) to allow us to collect and analyze their Weibo posts by computer programs. A total of 1,918 participants responded to the survey between June and September 2016. Among them, 1,629 allowed us to access their Weibo data. We used a custom Python script to automatically collect 198,485 Weibo posts from these 1,629 users. All the posts were posted from January 2009 to September 2016.

### Depression Symptom Screening Test

We used a depression screening test, namely, the Center for Epidemiologic Studies Depression Scale (CES-D), to infer participants’ depression symptom levels. The original CES-D was a 20-item self-reported scale designed to measure depression symptoms in the general public (28). We adopted the short form developed by Kohout (29) and translated into Chinese by Chin et al. (30). A back translation version of the short form is provided in Appendix A. This short form sacrifices little precision and taps the same symptom dimensions as the original CES-D. Both the original CES-D and the short form were tested in the Chinese population. The internal consistency of CES-D 10 in the Chinese population was satisfactory (Cronbach *α* = 0.78 ± 0.79). Reliability over a period of 3 years was also found to be significant (*r* = 0.44, p 50.01) (31). In the short form, participants were asked to rate the extent to which they experienced depression symptoms. An example item could be: “My sleep was restless.” Responses are on a Likert-type scale, including 1 (“Rarely or none of the time”), 2 (“1–2 days last week”), 3 (“3–4 days last week”), 4 (“Every day”), and 5 (“Every day for at least two weeks”). Appendix A shows the standard Chinese questionnaire used in this study (with English translation).

### Stressful Life Event Survey

We examined the stressful life events recently encountered by participants before completing the survey. A major problem in assessing life stress is that whether an event is considered stressful or not could be very subjective. Some stressors can be motivating to one person but stressful to others. Therefore, we focused on objective stressful life events. After asking the participants whether they had experienced, up to 3 months before completing the survey, any life events that they perceived as stressful, we asked them whether they had recently experienced one of three specific stressful events: relationship breakdown, a family member/close friend being diagnosed with severe illness, or being fired from work. The first two were ranked within the top three to top ten in the Social Readjustment Rating Scale (SRRS) (32); and the last one was found to drop from the top 10 to the top 50 in a recent revisit to SRRS (23). In addition to these specific events, we provided the option of “others” on the survey, which allowed participants to include any events that they themselves perceived as significant life stressors.

## Methodology

### Summary Statistics

**Figure 1:**
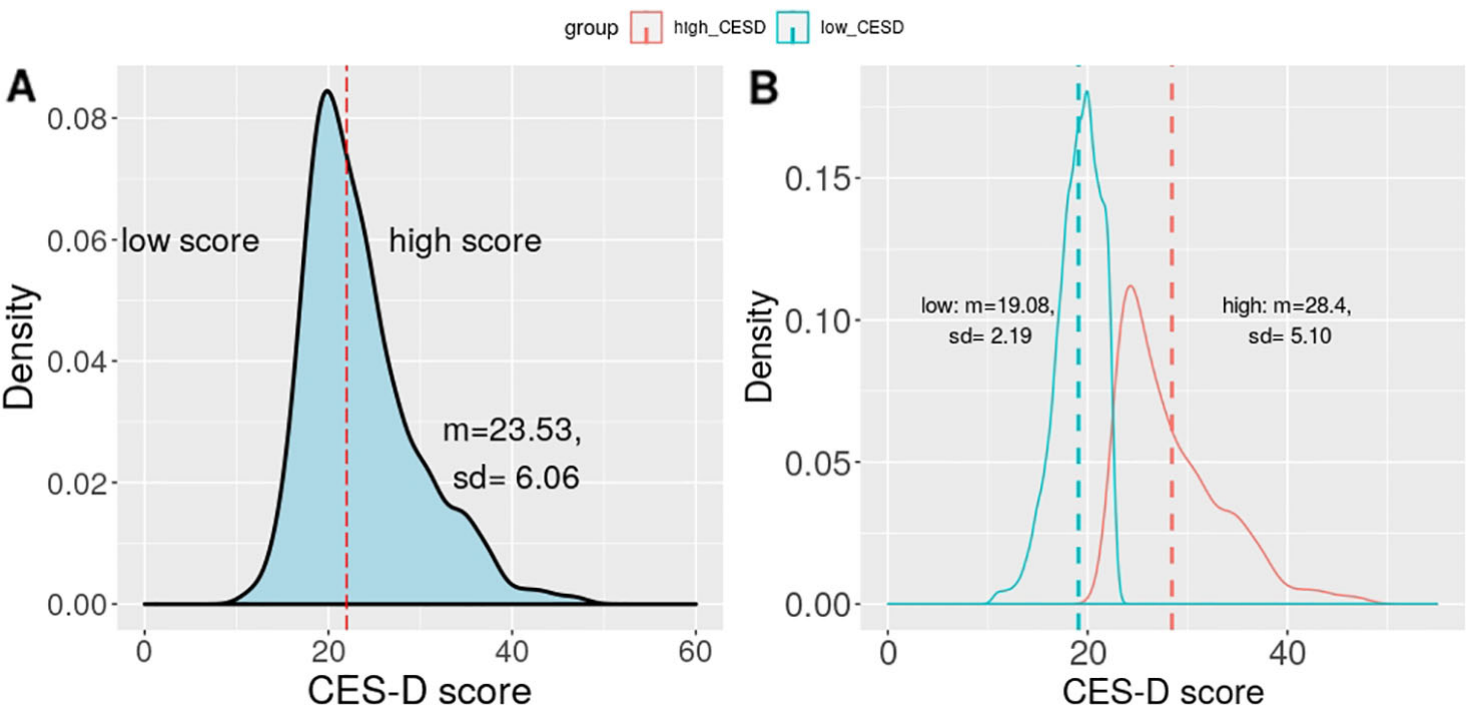
Statistics of the Center for Epidemiologic Studies Depression (CES-D) score. **(A)** shows the CES-D score in the whole sample (N = 1,628), the red line divides the users into the high and low groups according to their CES-D scores. **(B)** shows the CES-D scores of the high (N = 776) and low (N = 853) groups, the dotted lines indicate means.

To compare the patterns of affect score from participants with high level of symptoms with those with low level of symptoms, we split participants into two groups according to their CES-D survey scores using a cutoff point of 22, which has been applied in multiple studies (9, 28, 33). Here, we present the summary statistics of the two groups. **Figure 1A** shows the basic statistics of users’ CES-D scores, and **Figure 1B** shows the statistics in the H-group and L-group. Internal consistency of the CES-D scale was high (Cronbach’s *α* = 0.62). Participants posted more positive Weibo posts (*N*=50,731) than negative ones (*N*= 35,651). Their mean affect was negatively correlated with their CES-D scores (*r* = -0.13, *p <* 0.001).

### Computing Affect Score

We used a sentiment analysis service provided by Lexalytics (https://www.lexalytics.com/) to assign a continuous sentiment score ranging from 1 to −1 to each document (i.e., Weibo post). Lexalytics has performed satisfactorily compared with other popular sentiment annotation tools (34), such as OpinionFinder and Sentistrength. Lexalytics uses part-of-speech tagging to identify adjective-noun combinations, and then counts the number of affective words in a sentence. The algorithm adds weights to the word count according to a sentiment library developed by Lexalytics. The sentiment library contains an extensive collection of adjectives, each manually scored by human annotators according to their judgment of how negative or positive the word is. The sentiments of the words are inverted in the presence of negators (e.g., “not”) or some conjunction (e.g. “but” and “however”). Lexalytics also accounts for multilayered sentiment; if a sentence contains both positive and negative affective words, the two types of words may cancel each other out, thus making the document neutral. Before computing the affect score, we preprocessed the Weibo posts following some simple procedures, including removing Email addresses and hyperlinks and encoding emoticons using descriptive words within square brackets, e.g., [sad].

### Visualizing Affect Pattern

We visualized the affect pattern of each user over a time series in the unit of day, and applied a generalized linear model to smooth the time series. The timeline was aligned in a backward manner, with the day when users completed the CES-D scale as “Day 0” and the day before “Day 0” was “Day 1.” Note that “Day” here is not a calendar day. A calendar day might include events that influence public affect in general. For example, extreme weather might lead to more NA, and holidays to PA. To reduce the noise from holidays, weather, and other confounding factors on calendar dates, we residualized the daily affect of each participant *vi* by subtracting it with the mean affect score of the whole sample on that day *µ*. Therefore, the adjusted post affect score would be *vi* − *µ*.

**Figure 2:**
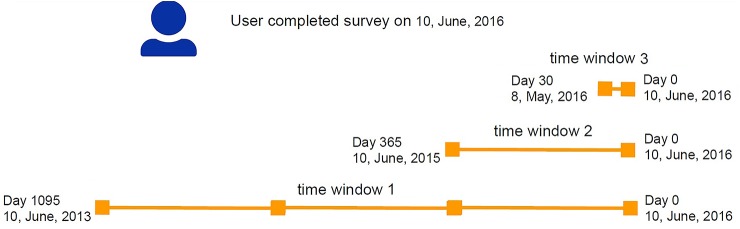
Time windows.

We were interested in participants’ affect patterns at different stages before they developed depression symptoms. Examining the stages presented a challenge: How to define the time window of each stage? Note that the self-reported score is not a gold standard for diagnosis, participants might develop the symptoms long before they completed the measurement scale. Therefore, we first defined the time window (T3) as Day 0 to Day 30 to observe the affect score while participants were experiencing high symptoms. Literature suggests that early symptoms happened in a time ranging between 6 weeks and 23 months (35, 36). Hence, we defined T2 as Day 0 to Day 365 to examine the development of early symptoms within a year. We were also interested in the affect pattern beyond the flare-up of symptom, so we set up T1 as Day 0 to Day 1095 to observe the longitudinal affect over the three years (see **Figure 2**). Note that depression is a persistent condition that can last for years if left untreated, thus, some participants might have been living with symptoms for years.

### Cognitive Vulnerability Analysis

Individuals with cognitive vulnerability are more likely to develop depression symptoms if experiencing a stressful life event. Therefore, we conducted a within-subject correlation analysis between affect scores and CES-D scores before the occurrence of specific stressful life events. We divided the participants into two groups based on whether they had experienced certain life events in the recent 3 months. We focused on examining the events that could bring more severe impact on participants’ depression symptoms. Among the 250 participants who answered the life event questions, 77 reported that they had encountered at least one stressful life event recently (CES-D median = 26) (see **Table 1**). Among them, those who reported having a breakup (CES-D median = 29) or being fired from work (CES-D median = 29) tended to develop more depression symptoms. Accordingly, we grouped the participants according to whether they had been through these two life events in the recent 3 months.

**Table 1:** Center for Epidemiologic Studies Depression (CES-D) scores of the participants who experienced stressful life events.

events	N	CES-D	
		Median	Q1–Q3
total	77	26.00	21.00–32.00
break up	33	29.00	24.00–34.00
illness	23	23.00	20.00–24.52
being fired from work	12	29.00	23.50–31.25
other	31	25.00	21.00–32.00
more than two	17	26.00	21.00–35.00

Since stressful life events occurred in the recent 3 months (90 days) prior to the completion of the survey, we selected three time windows: 90 days to 1 year, 90 days to 2.5 years and 90 days to 3 years before the completion of the survey. In each window, we computed the residualized daily mean affect score and conducted a correlation analysis between the mean affect score and CES-D score. Due to multiple correlation tests in the analysis, we used a permutation test to reduce the uncertainty of *p*. In the permutation test, the labels of the data were rearranged in each computation and the *p*-value achieved in the statistical test was estimated based on 20,000 simulations.

### Rumination Language

Before analyzing the characteristics of rumination language, we first annotated the posts that contained rumination language following one of the three stressful life events (c.f Section 2.4). We selected the Weibo posts from 77 participants who reported having encountered at least one stressful life event in the recent 3 months, and focused on their Weibo posts between Day 0 and Day 90, because this is the time window closest to the time point when these participants self-reported their depression symptoms. Instead of using a keyword approach to capture the stressful life events, we manually annotated life events. In some cases, we identify life stressors from contextual information. For example, a post such as “She left me, my heart is broken” indicates a relationship breakup.

Then, we analyzed whether these posts reflected any of the three types of cognitive tendencies (37), including the tendency to focus on negative attributions and inferences, the tendency to focus on hopeless thoughts, and the tendency to focus on coping strategies. To protect users’ privacy, we removed the name entities and other sensitive information that might reveal the identities of these persons from the Weibo examples.

Our annotation was carried out with an in-house, online annotation tool. Annotation guidelines for both rumination and stressful life events can be found in Appendix B. Two authors annotated life stressors. The interrater reliability was 0.80 for rumination language and 0.92 for life events. Appendix B shows the annotation guideline for both annotation tasks.

## Result: Cognitive Vulnerabilities

### Visualizing Affect Patterns in Multiple Time Windows

We computed the average affect score of each individual and examined the affect patterns in the three time windows. **Table 2** shows the statistics of the affect scores in the H-group and L-group, respectively. It is evident that the H-group consistently shows lower affect in T1, T2, and T3.

**Table 2:** Basic statistics of affect and Center for Epidemiologic Studies Depression (CES-D).

Time	H-group					L-group					all participants	
	N	affect		CES-D		N	affect		CES-D		affect	
		M	sd	M	sd		M	sd	M	sd	M	sd
T1	781	−0.009	0.11	27	24–34	861	0.01	0.09	27	24–34	0.00	1.32
T2	732	−0.002	0.07	19	18–20	678	0.006	0.06	19	18–20	0.002	1.55
T3	794	−0.02	0.05	27	24–34	500	0.03	0.06	19	18–20	0.005	2.22

M, mean; sd, standard deviation; N, number of participants in each time window; T1, three-year time window; T2, one-year time window; T3, 30-day time window; Affect, mean affect score on a day.

**Figure 3:**
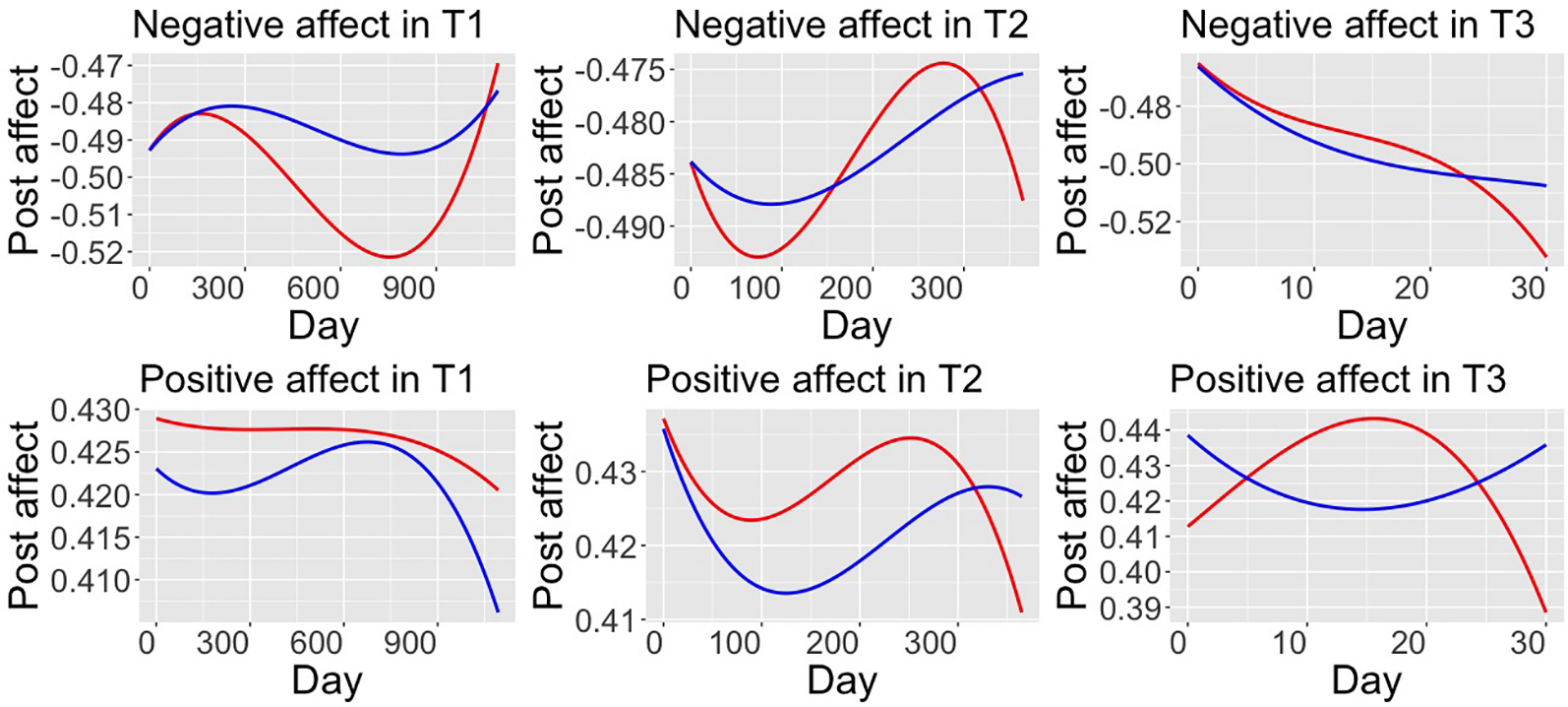
Affect patterns of the high symptoms and low symptoms group. Red lines, H-group; Blue lines, L-group. T1, three-year time window; T2, one-year time window; T3, 30-day time window. Figures of negative affect are negative up.

We plotted the participants’ affect against the time (see **Figure 3**). Note that Day 0 was the day when the participants completed the CES-D survey. We conducted a Welch t-test to detect the significance of the affect differences between the two groups, and the *p*-values were adjusted following the Bonferroni correction.

We found distinguished affect patterns between the two groups. As for NA, the H-group showed significantly higher NA than the L-group in T1 (3 years) (*t*(2044) = 4.65, *p <* 0.001, Cohen’s *d* = 0.20) and T3 (30 days) (*t*(58.6) = 3.45, *p <* 0.05, Cohen’s *d* = 0.88), but not in T2 (1 year) (*t*(724.7) = 1.05, *p >* 0.05, Cohen’s *d* = 0.13). As for PA, the H-group showed significantly higher PA than the L-group in T2 (*t*(721.3) = 1.98, *p <* 0.05, Cohen’s *d* = 0.14), but not in T1 (*t*(2046) = 1.76, *p* = 0.07, Cohen’s *d* = 0.08) and T3 (*t*(59.8) = 0.44, *p* = 0.6, Cohen’s *d* = 0.11).

The persistently high level of NA signals a negative cognitive bias in the H-group. The elevated level of PA might indicate that the participants in the H-group incorporated various coping skills to tackle life stress. However, this coping ability might be impaired when an individual has already developed severe depression symptoms.

### Life Stressors and Vulnerability

We first looked at how many participants actually mentioned on their social media posts the life stressors they reported to us. Our annotation result shows that only seven participants indicated a breakup experience, but no one mentioned being fired from work. Among the 23 participants who reported to us that their significant others were diagnosed with severe illness, only one mentioned it on Weibo. Our result suggests that participants are very selective regarding what life stressors they want to share to the public, of which, relationship breakup is the one most commonly mentioned on social media.

In the previous sections, we observed that people with high CES-D scores used more negative affective words on their social media posts over years, which indicates certain degrees of cognitive bias. In this part of analysis, we further raise the question, do people displaying cognitive bias in their social media posts over a long period of time have higher risks of developing depression symptoms when they are under stress?

To answer this question, we conducted a correlation analysis between affect and depression symptoms before the life stressor occurred. Since participants reported that life stressors happened between day 0 and day 90, we used day 90 as a pivot. We broke down the timeline into three time windows and observed the correlations between depression symptoms and social media affect in each time window (see **Table 3**). Here, the time line starts from the pivot to 1 year, 2 year and 3 years before the pivot. Participants in table 3 all completed the life event survey. The ‘Yes’ group refers to participants who reported a relationship breakdown or being fired from work (N = 45), the ‘No’ group (N = 205) refers to participants who experienced other life stressors or no life stressors. We divided participants according to life stressors they experienced because those who experienced a relationship breakdown or being fired from work have highest self-reported symptom score (see **Table 1**). We found that the amount of negative affective words in the ‘Yes’ group is moderately correlated with their self-reported symptom score long before the life stressor occurred. Participants in this group seem to be less satisfied with their daily life activities in general. Our result suggests cognitive vulnerability can be observed in longitudinal social media data.

**Table 3:** Correlation between Center for Epidemiologic Studies Depression (CES-D) score and social media affect before the occurrences of life stressors.

Relationship breakdown/fired from work	Yes			No		
time window (from 90 days to…)	1 year	2 years	3 years	1 year	2 years	3 years
positive affect	−0.17	−0.17	0.06	−0.017	−0.025	−0.025
negative affect	0.38	0.40*	0.40*	−0.038	0.021	0.021

‘Yes’ refers to the fact that the participants experienced relationship breakdown or being fired from work in the recent 3 months before they completed the CES-D measurement. The number shows the correlation between participants’ CES-D score and their average affect over a period of time (1 year, 2 years, 3 years before they completed CES-D measurement).

*p < 0.05; p < 0.1.

## Results: Stress-Reactive Rumination

Among the three life events we examined in this study, relationship breakdown was the most commonly mentioned stressor on Weibo. Therefore, we focused on examining the rumination language from the participants who had experienced relationship breakdown (N = 33). Among them, only seven mentioned the word “breakup” on their Weibo posts. We annotated 151 Weibo posts from the seven participants, these posts were posted between day 0 and day 90. We found that 23% (N = 33) of their posts contained rumination language, and all of the rumination contents were related to relationship breakup.

We observed that the rumination language indicated various types of cognitive tendencies, and the most common one was focused on negative attribute. People tend to ruminate on the loss of a relationship. For example, “I can’t face reality.” “I can’t move on.” “Your favorite Mr. Z has left you.” (see Examples 1 and 2 in **Table 4**). Occasionally, people have hopeless thoughts, such as “life is meaningless” (see Example 3 in **Table 4**). They tend to linger on the negative emotions, for example, “My tears keep pouring down when I’m not busy with anything.” Meanwhile, we also observed that people adopted various coping strategies, such as reappraisal, e.g., “I don’t like you anymore.” and problem-solving, e.g., “I have to treat myself well.” “stay strong and still” (see Example 4 in **Table 4**). These coping strategies often contain positive emotions.

**Table 4:** Examples of rumination contents.

Participant	Rumination content	Translation
Example 1: Focus on negative attribute		
1	你最爱的Z先生，已经离开你了	Your favorite Mr. Z has left you.
1	隐藏了关于你的一切，不后悔曾经爱过你，也没力气再向前。如果我的心痛全世界没有一个人懂，我也不后悔曾经爱过	I hide everything related to you, I never regret loving you, but I can’t dare to move on. Even if no one else in the world knows my sorrow, I will never regret I fell in love with you.
1	曾经最爱的那个人怎么就不爱了呢，想起曾经的快乐，和再也回不去的困惑，总是做不到头也不回的回到现实中去。总想着有一天春风和煦，我们还是可以一起离开这里，忘掉所有不愉快。可是改变了就是改变了啊	How come I don’t love the person I used to love anymore? I remember all the joy and confusion, I can’t face the reality. I am always thinking about that one day, we will leave this place together and forget all the sorrows. However, something has changed.
1	一闲下来眼泪就往上涌，都会过去的，会过去的	My tears keep pouring down when I am not busy with anything, and everything will be fine, will be fine.
Example 2: Focus on negative attribute		
2	可能你很久以后才学会爱人，我很遗憾只做了你途中の风景	Maybe you will learn how to love again, and it’s a shame that I’m only scenery in your life.
2	一万次的道别难道还不够，也许再见只是一个承诺，你在夕阳里回首的轮廓，我到现在依然记得	No matter how many times we say farewell, it’s never enough. Maybe this is just a promise to you. I still remember the way you look at me at sunset.
Example 3: Focus on hopeless thoughts		
2	一句话也不想说，不是淡薄而是呵呵，不是不多想而是乏了。静的只听得见自己的呼吸，淋一场大雨不管不顾，放声大哭。然而眼泪也出不来，没有意义没有寻找的生活，人早已经麻木。在这么下去得抑郁不可	I don’t want to say anything, because I’m too tired to think of anything else. The world is so quiet that I can hear my breath. I showered in the rain and cried out loud. There are no tears in my cry, and life is meaningless, I feel numb about my life. I will be depressed soon.
Example 4: Focus on coping strategies		
3	不做白日梦了，认真做事，好好生活	Stop daydreaming, work hard and enjoy life. (problem solving)
3	终于可以不再爱你了，真好	Finally, I don’t love you anymore, that’s great. (reappraisal)
3	25岁，不矫情，不任性，不抱怨！摒弃外界眼光，只为自己而活。	I’m 25 years old now, and I’ll be strong, mature and stop complaining. (problem solving)
3	自己也要对自己好啊	I have to treat myself well. (problem solving)
2	真正能依靠的唯有内心的强大，坚强难得，却定心	I should rely on the power inside me, stay strong and still. (problem solving)
2	其实真的不用那么敏感，我不在喜欢你了，我的名字只是来自一句歌词，没翅膀也做梦想家	Don’t be over-sensitive, I don’t like you anymore. My name comes from a song lyric: “I want to be a dreamer even without wings”. (reappraisal)

## Discussions

### Implications

In this paper, we applied a data-driven approach to analyze individuals’ affect patterns on a Chinese social media platform. Overall, we found that people’s affective expressions on social media could reflect their risks of developing depression symptoms long before early symptoms were expressed. Therefore, researchers should examine social media posts over a longer time frame when studying depression symptoms.

By looking at NA and PA separately, we found that individuals with high depression symptoms tended to use more negative and positive affective words on their social media status updates in general. This finding is in contrast with the findings in traditional empirical studies but aligns well with the recent findings from Locatelli and colleagues (19). We speculate this is related to the fact that social media users tend to present themselves positively (20, 21). This finding might suggest that users with high depression symptoms are more likely subject to a greater level of self-presentation bias. Accordingly, researchers should take into account the characteristic of specific social media behaviors while using social media data to study psychological symptoms.

We also found that users rarely mentioned significant life stress on social media. Among 77 people who told us on our survey that they had experienced a stressful life event, only about 10% of them had mentioned it on a Weibo post. This encourages the researcher to be aware of a highly biased sample when conducting research on life stressors with social media data. Since female users are more likely to disclose their mental state (27), our sample for rumination might also be biased toward female users.

So far, most of the existing studies that make use of social media data to infer depression symptoms have only used a quantitative approach to analyze the language in the posts. Few studies have attempted to examine the content that is directly linked to negative cognitive biases. In our study, we examined the rumination language from the participants who had recently experienced a breakup. This group of people also had exceptionally high symptom scores (M = 29). We found that 23% of their Weibo posts contained rumination contents. Our finding aligns well with the literature on depression symptoms and post-event rumination (38, 39). Although their rumination often focused on negative attributes, we also found evidence of problem-solving coping strategies (40). These findings provide insights into identifying social media content that is directly associated with depression symptoms, and call for a more calibrated approach to measure depression symptoms by looking at cognitive biases in social media data.

### Limitations

Chinese Weibo has a sophisticated filtering system to censor Internet data; contents considered “harmful” to the community will be immediately tagged and discarded (41). Hence, swear words and some negative opinions are often censored in such a social media platform. In order to evade the censorship, social media users start to use metaphorical language or change the written forms of swear words. Simple natural language processing techniques are less reliable in detecting such variations of negative or sarcastic expressions. In addition, there are confounding factors that might affect our conclusions, such as the offline behaviors not observable in social media data. Therefore, affect expressed on social media data only reflects a small portion of daily life affect. Furthermore, the results of this study are also biased toward the data generated by active Weibo users. All these limitations prevent us from making stronger or more general claims, but our study still provides useful insights about cultural dependent symptoms and vulnerability as indicated by social media data.

## Conclusions and Future Work

We presented a comprehensive study of negative and positive affects shown on the Weibo posts of Chinese social media users. First, we collected Weibo status updates from users who completed a survey to measure their depression symptom levels and detect their life stressors. We visualized users’ social media affect in a temporal manner and proceed to examine their language after they experienced specific life stressors. Our results show that increased negative and positive affects in social media status updates are closely related to elevated depression symptoms. Such a unique pattern reflects cognitive vulnerability to developing depression symptoms. Users with cognitive vulnerability have higher depression symptom scores after they experience specific stressors in life. Finally, we proposed to study the rumination language in social media content with negative affect, because rumination language is associated with dysfunctional stress response.

This study reveals how social media based measures serve as a longitudinal resource to monitor participants’ vulnerability to mental problems. It is potentially useful for clinicians to identify individuals at risk. Some of the findings could be limited to Chinese culture. More cross-cultural studies are necessary to identify the cultural differences and their influences on mental disorders.

## Data Availability Statement

The data of this paper can be made available based on appropriate requests to the corresponding authors.

## Ethics Statement

This study was approved by the College Research Ethics Committee of City University of Hong Kong. The methods were carried out in accordance with the approved guidelines from the College Research Ethics Committee. Written informed consents were obtained from all the participants.

## Author Contributions

LC contributed to the design and data collection. LC and TG contributed to data analysis and interpretation, drafting, and revising the paper. CC contributed to supervision and paper revision.

## Funding

TG is supported in part by the Natural Science Foundation Committee of Guangdong Province (Grant No. 2018A0303130235) and the MOE Project of the Centre for Linguistics and Applied Linguistics, Guangdong University of Foreign Studies.

## Conflict of Interest

The authors declare that the research was conducted in the absence of any commercial or financial relationships that could be construed as a potential conflict of interest.

## Appendix A Survey Questions

**Introduction:** This document contains the survey questions and the translated/back translated version of the CES-D survey.

**Table:**

Data Collection Consent Form		Options
本人是香港城市大学心理系硕士生，此研究目的是用微博数据建计算机模型自动预测心理特质。我们需要搜集你的微博数据，如同意我们收集您的数据，请在本问卷填写您的微博用户名称。如不同意，请忽略。本问卷收集的数据只用于研究用途。参与研究同意书:		1. 本人已经明白上述的资料，并同意参与这次研究。2. 不同意参与这次研究
I am a master student from the City University of Hong Kong, HKSAR. We are looking for Weibo users to complete this survey. The purpose of this study is to identify the characteristics of social media users with depression symptoms. You are opting to fill out the review below and allow us to collect your Weibo data. Your data will only be used for this study.		1. I understand the information, and I agree to participate in this study. 2. I don’t want to participate in this study.
Life Events Survey		
Question	Translation	Options
1. 你的微博名称（不是你邮箱的名称，例如：luciasalar) 最关键的一步，大家务必要填写。实在没有微博请填写”无”	Your Weibo account (Not your email address, e.g.: luciasalar)	
1. 你过去三个月有经历什么重大压力吗？（例如：失恋，亲人患病)	Have you experienced any major stress in the past 3 months? (e.g., breakup, family member being diagnosed with illness)	(a) Yes (b) No
2. 如选择有，请选择（可多选）：	If you selected Yes in the previous question, please specify the stressful life event (multiple choice):	(a) breakup (b) family member being diagnosed with severe illness (c) being fired from work (d) Others
3. 你曾经得过抑郁症吗？	Have you ever been diagnosed with depression?	(a) Yes (b) No (c) I don’t know

Below is the translated CES-D scale and its back translation, the answers for each questions are: a. I barely feel that way; b. 1-2 days last week; c. 3-4 days last week; d. every day; e. every day and it has been for 2 weeks.

**Table:**

Question	Back Translation
1. 你最近不想吃东西、胃口不好	You have a poor appetite recently and you don’t want to eat anything.
2. 你觉得心情很不好	You feel like having a bad mood.
3. 你觉得做事情很不顺利	You feel that things don’t go through smoothly
4. 你睡不安稳	You have an uneasy sleep
5. 你觉得很快乐	You feel happy
6. 你觉得人人都不友善	You feel others are not friendly
7. 你觉得日子过得很好很享受人生	You are enjoying your life
8. 你觉得人不喜欢您	You think that people don’t like you
9. 你提不起劲做任何事	You don’t have a mood to work on anything
10. 你觉得很悲哀	You feel sad
11. 你觉得寂寞，孤单	You feel lonely

## Appendix B Annotation Guideline

**Introduction:** This document describes the annotation guidelines for marking up Weibo that indicate specific stressful life events and ruminating behaviors. The document contains two tasks. Task 1: Annotate stressful life events, including relationship problem, unemployment and severe illnesses. Task 2: Annotate rumination in the post.

**Task 1: Annotate stressful life events.** In this task, you are provided three sets of posts from Weibo users who have reported that they experienced at least one of the following LIFE EVENTs: Relationship breakdown, unemployment, and severe illness of a friend or family member. The title of the page indicates which LIFE EVENT that the author had been through.

How to Annotate LIFE Events

1. Relationship breakdown. In this task, please annotate the earliest post that indicates that the poster experiences a breakup, divorce, or loss of a relationship. You only need one label for each user in this task. Once finding a post as such, you can skip the rest of the posts from the same user.
2. Unemployment. In this task, please annotate the earliest post that indicates the state of unemployment. You only need one label for each user in this task. Once finding a post as such, you can skip the rest of the posts from the same user.
3. Severe illness. In this task, please annotate the earliest post that indicates a family member or close friend being diagnosed with terminal illness. You only need one label for each user in this task. Once finding a post as such, you can skip the rest of the posts from the same user.

**Task 2: Annotate rumination in the post.** Rumination is repetitively going over a thought or a problem without completion. The ruminating behavior on Weibo should be the act of posting multiple Weibo that show deep thoughts or reflections about the same topic. Content of ruminative thought can be positive or negative.

Please select RUMINATION if the post fulfill all of the following conditions:

1. Author describes a specific thought, emotion or a problem in the post.
2. The thought or problem reoccur within one week after the previous post (Read the timestamp).

Here shows an example of rumination on relationship breakup:

1. 可能你很久以后才学会爱人，我很遗憾只做了你途中の风景。Timestamp: 2015-2-14 (Translation: It might take longer for you to learn how to love a person. It’s a shame that I am just scenery in your life.)
2. 有时候会想，为什么偏偏是天籁。以后每次坐他的车，都难以忘记第一次你用挂挡の那只手牵着我，跟我说手动挡都无所谓，何况这是自动挡。真的希望回忆不能欺骗时光太久，愿无岁月可回首，愿无深情共白头。Timestamp: 2015-2-15 (Translation: I can’t forget how he held my hand the first time I was in his car. I hope my memory does not lie to the time for too long. Time flies by, and I hope we can grow old together.)
3. 隐藏了关于你的一切，不后悔曾经爱过你，也没力气再向前。如果我的心痛全世界没有一个人懂，我也不后悔曾经爱过。Timestamp: 2015-2-15 (Translation: I hide everything about you. I never regret to fall in love with you. But I can’t move on anymore. Even if no one can understand how painful I am, I will never regret being in love with you.)

All the above examples describes feelings toward the breakup and or memory toward the ex-lover and these posts occur within a few days.
